# Insulin resistance and coronary artery disease in non-diabetic patients: Is there any correlation?

**DOI:** 10.22088/cjim.9.2.121

**Published:** 2018

**Authors:** Jamshid Vafaeimanesh, Mahmoud Parham, Samieh Norouzi, Parinaz Hamednasimi, Mohammad Bagherzadeh

**Affiliations:** 1 Gastroenterology and Hepatology Disease Research Center, Qom University of Medical Sciences, Qom, Iran.; 2 Clinical Research Development Center, Qom University of Medical Sciences, Qom, Iran.; 3 Students’ Research Committee, Qom University of Medical Sciences, Qom, Iran.; 4 Gastroenterology and Liver Disease Research Center, Iran University of Medical Sciences, Tehran, Iran.

**Keywords:** Insulin resistance, Coronary artery disease, Non-diabetic patients

## Abstract

**Background:**

Cardiovascular diseases are the most common causes of death in the world and type 2 diabetes is one of them because it is highly prevalent and doubles heart disease risk. Some studies suggest that insulin resistance is associated with coronary artery disease in non-diabetics. The aim of this study was to evaluate the association of insulin resistance (IR) and coronary artery disease (CAD) in non-diabetic patients.

**Methods:**

In this cross-sectional study, from September 2014 to July 2015, 120 patients referring to Shahid Beheshti Hospital of Qom were evaluated. Their medical history, baseline laboratory studies, BMI and GFR were recorded. After 8 hours of fasting, blood samples were taken from the patients at 8 am, including fasting glucose and insulin level. We estimated insulin resistance using the homeostatic model assessment index of IR (HOMA-IR). Finally, we evaluated the association between IR and CAD.

**Results:**

Totally, 120 patients were assigned to participate in this study, among them, 50 patients without CAD and 70 with coronary artery stenosis. Insulin resistance (HOMA-IR> 2.5) was positive in 59 (49.3%) patients and negative in 61 (50.7%) patients. Hence, the correlation between IR and CAD was not statistically significant (P=0.9).

**Conclusions:**

In this study, although the correlation was not found between insulin resistance and coronary heart disease, among men, we found a significant association between coronary heart disease and insulin resistance.

Cardiovascular diseases are the most common causes of death in the world (1) and type 2 diabetes is one of them because it is highly prevalent and doubles heart disease risk (2, 3). Accordingly, studies have shown that increased glucose and insulin concentrations can be pro-atherogenic (4, 5). Since all type 2 diabetic patients are insulin resistant, it is difficult to assess its role in the development of coronary artery disease of diabetics. Based on animal studies, insulin resistance is exclusively involved in the early and advanced stages of atherosclerosis, and hyperglycemia plays an important role in the early stages of atherosclerosis (6). In addition, it seems that insulin resistance modifies the effect of insulin on the vascular wall and has anti-atherogenic effect in the insulin sensitive state and pro-atherogenic effect in the insulin resistant state (5). Also, some studies showed that among those with type one diabetes who have higher rates of insulin resistance, the risk of coronary disease is higher (7, 8); so, the question is apart from changes in serum glucose level, does increased insulin resistance play a role in the development of atherosclerosis independently? High insulin and glucose concentrations are direct consequences of insulin resistance. Insulin resistance can promote the development of atherosclerosis through increased glucose and insulin concentrations and also mechanisms that involve dyslipidemia, hypertension, and inflammation (4, 6).

Furthermore, procoagulant state inflammation, endothelial dysfunction, hyperuricemia, enhanced sympathetic, nervous system activity, increased renal tubular sodium reabsorption, increased incidence of obesity and acute phase proteins, changes in adiponectin levels, interference in the synthesis of NO are the other contributing factors (9-12). Therefore, cardiovascular diseases may be a consequence of insulin resistance rather than being caused by toxic effects of high insulin or glucose concentrations. Homeostasis model assessment insulin resistance (HOMA-IR) is a validated and commonly used marker of insulin resistance which incorporates both glucose and insulin concentrations and represents insulin resistance that can promote atherosclerosis through several mechanisms (4, 6). Some studies suggest that insulin resistance is associated with coronary artery disease in non-diabetic subjects (9, 13). This relationship is interesting because insulin resistance is increasing nowadays and if the studies prove the association between IR and CAD, medication and lifestyle changes will decrease insulin resistance, then coronary artery disease can be prevented. The aim of this study was to evaluate insulin resistance and coronary artery disease in non-diabetic patients.

## Methods

**Patients:** This cross-sectional study was performed from September 2014 to July 2015. The inclusion criteria were: patients who were candidates for coronary angiography in the catheter Lab of Shahid Beheshti Hospital of Qom University of Medical Sciences. From all of the subjects, their medical history was obtained and recorded, body mass index (BMI) was calculated as weight in kilograms divided by the square of the height in meters (kg/m2) and blood samples were taken for laboratory to be studied.

Patients with history of CHD or prior coronary revascularization, heart failure or any type of cardiomyopathy, familial hypercholesterolemia, impaired liver or renal function, anemia, malignancy and diabetes were excluded from the study.

**Laboratory studies and the assessment of insulin resistance:** After an 8-hour fasting, blood samples were taken from the patients at 8 am, including fasting glucose, insulin concentration, CBC, liver function tests, lipid profile, BUN and creatinine. All parameters were evaluated in the hospital laboratory. We estimated insulin resistance using the homeostatic model assessment index of IR (HOMA-IR) developed by Mathew with the following formula: HOMA-IR = baseline insulin concentration (mU/L)×baseline glucose concentration (mg/dL)/405 (14).

**Coronary angiography** ***: ***Coronary angiography was performed by femoral artery using Judkins method (15). Two experienced cardiologists unaware of the patients’ enrollment reviewed all angiograms. If they did not have the same view, the third cardiologist would see the angiographic film and then, based on angiographic results, patients were divided into two groups with and without coronary artery disease. Coronary artery disease was defined as more than 50% luminal diameter stenosis of at least one coronary artery. Patients with CAD were divided into two groups, single and multi-vessel disease (SVD and MVD). Patients without CAD were divided in two subgroups,group one had luminal diameter narrowing <50% and considered as minimal coronary artery disease (MCAD) and group 2 without luminal narrowing considered as normal.

**Statistical analysis:** Chi-square test was used for comparing between categorical data and correlation between them. These data are presented as numbers (percentages).We applied student's t-test to compare the continuous variables and were expressed as mean±SD. Differences between more than two groups were tested with one-way ANOVA. We also used multivariate logistic regression to control the confounding factors that were effective on the relationships between IR and CAD. Finally, we analyzed the data using SPSS 16.0, and a p-value less than 0.05 was considered statistically significant.

**Ethics:** All individuals signed informed consent prior to their enrollment in the study. Also, the study was planned according to the ethical guidelines of the Declaration of Helsinki and the approval of Ethics Committee of Qom University of Medical Sciences.

## Results

In this study, 50 patients were assigned to group without CAD, 16 (13.5%) patients had normal angiography and 34 (28.5%) subjects had minimal coronary artery disease (MCAD).70 patients were in CAD group of whom 26 (21.9%)subjects had single coronary artery stenosis (SVD) and 44 (36.5%)participants had more than one coronary artery stenosis (MCAD). Characteristics of both groups are shown in table 1.

**Table1:** Clinical and laboratory characteristics of both groups

**Parameter**	**CAD+**	**CAD-**	**P-value**
Age (years)	58.30±12.03	54.27±12.80	0.051
Male/female	42/28(60.1/39.9%)	22/28(44.2/55.8%)	0.008
Fasting glucose (mg/dL)	100.87±13.50	99.43±13.65	0.377
BMI (kg/m2)	27.16±4.17	27.11±4.00	0.914
Total cholesterol (mg/dL)	158.27±38.85	149.76±41.84	0.077
HDL cholesterol (mg/dL)	44.12±10.83	48.79±14.61	0.002
Triglycerides (mg/dL)	160.54±93.03	145.82±88.22	0.178
LDL cholesterol (mg/dL)	82.85±34.67	76.72±39.92	0.166
Systolic blood pressure (mmHg)	132.22±21.48	130.55±21.32	0.515
Diastolic blood pressure (mmHg)	80.32±12.67	80.21±10.12	0.939
White blood cell	7684.88±2422.63	7172.67±2160.02	0.065
Platelet	233250.00±61781.89	234233.33±59730.30	0.893
Insulin level (mU/L)	15.56±18.19	13.50±12.91	0.288
HOMA-IR	3.89±4.63	3.3847±3.50	0.06
HOMA-IR>2.5	43(61.4%)	23(46%)	0.232

CAD: coronary artery disease. BMI: body mass index. HDL: high density lipoprotein. LDL: low density lipoprotein.

HOMA-IR: Homeostatis model assessment index of Insulin resistance

* Values are presented as mean±SD except where otherwise indicated

Totally, 66 (55%) patients had insulin resistance (HOMA-IR> 2.5) and 54 (45%) patients did not have insulin resistance. As shown in table 2, the association between insulin resistance and coronary artery disease was not statistically significant. We also controlled the confounding effect such as, age, gender, hypertension and hyperlipidemia, using multivariate logistic regression and the same results between IR and CAD were obtained. To evaluate the differences of insulin resistance and coronary artery disease severity (between four groups including, normal, SV MCAD and MVD) using ANOVA test, this difference was not statistically significant (P=0.42, F=0.95). In addition, in the post hoc tests, no significant difference was found between the groups (table 3).

**Table 2:** Association between insulin resistance and coronary artery disease severity

**Confounding** **Factors**	** Severity** **Insulin resistance **	**Normal**	**MCAD** *	**SVD** **	**MVD** ***	**P-value**
With	No	8	19	9	19	0.9
	Yes	8	15	17	25	
Without	No Yes	8 8	19 15	9 17	19 25	0.65

Data are presented as number

* MCAD: minimal coronary artery disease

** SVD: single vessel disease

*** MVD: multiple vessel disease

**Table 3:** Relationship between insulin resistance and coronary artery disease

**CAD group**	**HOMA-IR**	**F**	**P-value**
Normal	3.10±2.40	0.95	0.42
MCAD*	3.50±3.90		
SVD**	3.40±2.80		
MVD***	4.20±5.40		
Total	3.70±4.20		

Data are expressed as mean±SD

* MCAD: minimal coronary artery disease

** SVD: single vessel disease

*** MVD: multiple vessel disease

## Discussion

In this study, 120 non-diabetic cases were evaluated and 53.5% were males. Although an association was found between CAD and IR, more detailed analysis had notable findings. For example, the association between CAD and IR was found in non-diabetic men. This finding was not statistically significant in women. The average age of the population was 57.8±12.69 years and 60% of them were 50-70 years old. This is similar to Karrowni et al.'s study that showed the higher prevalence of CAD in the elderly and most of the patients were in the same age group (16). Although age is an important factor in CAD and IR, Kim et al. showed that age increase plays a role in elevating insulin resistance (17). In Schmiegelow et al.'s study, insulin resistance measures did not improve CVD risk discrimination and reclassification in postmenopausal women (18). In our study, CAD was more prevalent in patients over 70 years, while most IR was seen between 50-70 years. 

The reason for this finding is that people prone to type II diabetes whose disease have not appeared yet are in this group and the majority of people older than 70 years and with insulin resistance have been excluded from the study. In our study, the association between IR and CAD has not been achieved in terms of age. Other notable finding in our study was that unexpectedly the association between IR and CAD was found in normal BMI population which accounts for about one third of the patients. However, Karrowni et al. whose study was similar to us found no association between BMI and insulin resistance (16). Although Schauer et al. had a more accurate way of measuring insulin level (3-phasehyperinsulinemic or euglycemic clamp method) in their study. They have achieved similar results and found no association between BMI and insulin resistance (19). Insulin resistance has pro-atherosclerotic properties. In insulin resistance situation, the anti-inflammatory and anti-sclerotic properties of NO secreted by the endothelium is inhibited. This occurs due to decreasing receptors and change in the phosphoinositide 3-kinase pathway (20).

Although mitogen-activated protein kinase inhibition in endothelial cells and smooth muscle decreases the pro-atherogenic properties, this effect blocks the atherosclerosis pathway in mice by starting insulin (21, 22). Moreover, insulin receptors are located on monocytes and macrophages which promote the process of plaque formation during insulin resistance with some intracellular changes (23). In addition, reducing spherocytosis cells which collect the necrotic cells when necrotic cells increased worsens the sclerosis situation (24). Some studies have also mentioned the dependence of IR and hs-CRP which indicates inflammation deficiency of insulin (25).

In our study, the most available factor to assess the inflammatory role of IR was WBC. This is why we evaluated it. The mean WBC was 7471.46±2326.73 and 85% of the population were within the normal range and the highest IR and CAD rates were seen in this group. No association was found between IR and CAD based on WBC. To investigate the role of insulin on clotting factors, platelets have been used as the most accessible factor. The mean platelet level was 233659.73±60832.34 and 92% of the patients did not have normal platelet level and the highest IR and CAD rates were seen in this group. Varol et al. found that the mean platelet volume which is used as an indicator of platelet activity increases in patients with CAD in terms of insulin resistance (26). This association was not found in our study. The association between IR and CAD has been reported in the normal PLT category (P=0.04).In our study, almost half of the patients had insulin resistance (HOMA-IR> 2.5) and 58.3% had CAD on angiography.

The association between IR and atherosclerosis has been evaluated in some studies. For example, Karrowni et al. examined the association between IR and the extent of coronary atherosclerosis determined by angiography in 1073 non-diabetic post myocardial infarction patients and demonstrated an independent association between IR and multi-vessel coronary artery disease (16). In Granér et al.’s study, patients with more severe degrees of IR had a more severe, extensive, and distal types of CAD than patients with lower degrees of IR (27). Baseline fasting hyperinsulinemia could be a good predictor of significant coronary atherosclerosis in non-diabetic patients, which enables a more elegant cardio metabolic risk assessment in the setting of everyday clinical practice (25). Several large population-based studies have shown that hyperinsulinemia, a surrogate marker for insulin resistance, predicts incident CHD (28-30).

We neither were able to detect the overall association between IR and CAD, nor other studies found this association. For example, Vonbank et al. found no difference in HOMA-IR between subgroups of 986 consecutive patients undergoing elective coronary angiography and were divided according to the presence or number of lumen narrowing of ≥50%, irrespective of diabetes status (31). Accordingly, the lack of a positive association between angiographic CAD and HOMA-IR was also reported in an adequately powered study and strengthened our negative findings (31). Furthermore, a negative result had been previously described by Solymoss et al. who related the number of coronary arteries with ≥50% stenosis to fasting insulinemia (32). Gotoh et al. suggested that IR increases the risk of incident CVD through metabolic syndrome (33).

Though An et al. identified HOMA-IR as an independent predictor of one-year coronary atherosclerosis progression in CAD patients, however, Gensini score at baseline did not correlate to HOMA-IR, which was in agreement with our results (34). However, we demonstrated the association between insulin resistance and CAD in non-diabetic men in our study. This association provides a window that the pharmaceutical and behavioral interventions leading to a decrease in insulin resistance, may prevent coronary artery disease, particularly in high risk patients. For example Sugamura et al. suggested that adding Pioglitazone to successful statin therapy significantly improves insulin resistance and may be an effective therapeutic strategy for patients with CAD (35).

In conclusion, this study although the association was not found between insulin resistance and coronary heart disease, among men between coronary heart disease and insulin resistance, a significant correlation was found. Based on these findings, we recommend to rule out coronary heart disease in men with insulin resistance.
